# Efficacy and safety of topical statins for porokeratosis: a systematic review and practice-guided synthesis

**DOI:** 10.1093/skinhd/vzag043

**Published:** 2026-06-09

**Authors:** Mohammed Shanshal, Aarthy Uthayakumar, Emily Moon, Laksha Bala

**Affiliations:** Department of Dermatology, Imperial College Healthcare NHS Trust, Imperial College London, London, UK; Department of Dermatology, Imperial College Healthcare NHS Trust, Imperial College London, London, UK; Department of Dermatology, Imperial College Healthcare NHS Trust, Imperial College London, London, UK; Department of Dermatology, Imperial College Healthcare NHS Trust, Imperial College London, London, UK

## Abstract

**Background:**

Porokeratosis comprises keratinization disorders with few consistently effective, nondestructive treatments. Topical inhibition of the mevalonate pathway with statins is biologically plausible, but the clinical evidence is scattered across small reports.

**Objectives:**

To consolidate fragmented data into a pragmatic framework for clinicians and to highlight priorities for future controlled trails.

**Methods:**

We performed a PRISMA-based systematic review of PubMed, Embase, Scopus and ClinicalTrials.gov to 8 December 2025, including all study designs that reported patient-level outcomes with topical statins.

**Results:**

Thirty-three studies involving 162 patients met the inclusion criteria: three small, randomized trials and predominantly case series and case reports. In nonrandomized studies with analysable data (*n* = 98), most patients achieved partial improvement (*n* = 67/98 68.4%), whereas complete or near-complete clearance was less frequent (*n* = 16/98; 16.3%); disseminated superficial actinic porokeratosis (DSAP) appeared most responsive. Randomized evidence showed clinical improvement with topical statins but did not support routine addition of cholesterol to statin formulations. Topical statins were generally well tolerated, with several patch test-confirmed cases of simvastatin allergic contact dermatitis but no serious systemic adverse events. Certainty of evidence (GRADE) was low for DSAP improvement and serious harms, and very low for other subtypes, reflecting small sample sizes, risk of bias and heterogeneity.

**Conclusions:**

Overall, topical statin monotherapy emerges as a mechanism-based, field-directed option for porokeratosis, particularly DSAP, most often yielding partial rather than complete clearance with a favourable safety signal.

## Teaser

Topical statins are a mechanism-based, field-directed option for porokeratosis, but evidence has been scattered across small reports. In this PRISMA-based systematic review of 33 studies (162 patients), we harmonize outcomes and show that most patients, particularly with disseminated superficial actinic porokeratosis (DSAP), achieve partial rather than complete clearance, with a favourable safety signal and no consistent added benefit from cholesterol. The review provides a pragmatic framework to guide statin use and future trials in porokeratosis.

What is already known about this topic?

- Porokeratosis is a clonal keratinization disorder with limited nondestructive treatments and a recognized risk of keratinocyte cancer.
- Mevalonate pathway mutations provide a rationale for topical statins, and early case reports and small trials suggest benefit, especially in disseminated superficial actinic porokeratosis (DSAP).
- However, evidence is scattered, outcome definitions vary, and the incremental value of cholesterol add-on or long-term safety remain unclear.

What does this study add?

- This PRISMA-based, PROSPERO-registered review synthesizes 33 studies (162 patients) of topical statins to treat porokeratosis across subtypes and designs.
- It harmonizes patient-level outcomes, showing partial improvement in around two-thirds of patients and complete or near-­complete clearance in a minority, with DSAP best characterized.
- Randomized data indicate no consistent added benefit from cholesterol, a generally favourable tolerability profile and clear priorities for future controlled trials and maintenance studies.

Porokeratosis comprises a clinically and genetically heterogeneous group of disorders of clonal keratinization, unified histopathologically by the presence of cornoid lamellae. Disseminated superficial actinic porokeratosis (DSAP) is the most common subtype, typically presenting in middle-aged or older adults with annular, scaly lesions on sun-exposed sites, especially the lower extremities.^1^ Although traditionally considered benign, DSAP and linear porokeratosis (LP) carry a documented malignant transformation risk of approximately 1–10% in some series, most often to squamous cell carcinoma.^2–4^

Treatment options remain limited, and responses are often suboptimal. Topical 5-fluorouracil, calcipotriol, imiquimod and retinoids may induce partial clearance but are frequently associated with irritation and relapse.^5,6^ Procedural interventions such as cryotherapy, laser ablation or photodynamic therapy may be effective for isolated lesions but are less practical for extensive disease and carry a risk of scarring or dyspigmentation.^7,8^ There are currently no approved systemic treatments, and recurrence after discontinuation is common.^6^ Consequently, there is a practical need for effective, well-tolerated, non­destructive medical therapies that can be used over large body surface areas and maintained long-term. Topical inhibition of the mevalonate pathway (e.g. simvastatin and lovastatin) offers a mechanism-based option, but clinical reports are dispersed across small, randomized trials and uncontrolled series with heterogeneous outcomes, leaving clinicians without clear guidance.

Recent molecular insights implicate heterozygous variants in mevalonate pathway genes, most commonly *MVK*, *PMVK*, *MVD* and *FDPS*, in familial and sporadic porokeratosis. These defects reduce epidermal cholesterol and lead to accumulation of mevalonate-derived isoprenoid intermediates, disrupting keratinocyte differentiation.^9–12^ Topical statins inhibit 3-hydroxy-3-methyl­glutaryl coenzyme A (HMG-CoA) reductase upstream in this pathway, decreasing the build-up of proinflammatory isoprenoid intermediates and supporting a mechanism-based treatment approach for porokeratosis.^9–12^

The objectives of this review were to systematically synthesize clinical evidence on the efficacy, durability and safety of topical statins (alone or with cholesterol/adjuncts) for poro­keratosis; harmonize outcomes (complete/near-complete, partial, poor/no response); stratify by subtype; and appraise risk of bias. A formal synthesis is timely because three randomized datasets and multiple small series now exist, but results are scattered and reported with nonstandard outcome measures. We prespecified analyses to evaluate whether partial responses predominate (particularly in DSAP) and whether adding cholesterol provides any consistent incremental benefit over statin monotherapy.

## Materials and methods

### Protocol and registration

This was a prospective protocol study (PROSPERO: CRD420251123792), conducted and reported as per PRISMA 2020 guideline (https://www.prisma-statement.org/prisma-2020).

### Data sources and search strategy

We searched PubMed, Embase (Ovid) and Scopus from inception to 8 December 2025. No study-design filters were applied; in Embase we restricted the search to human studies written in English. Search terms combined a parent porokeratosis concept (porokeratosis; and in Scopus, porokeratos* using the ‘*’ wildcard) with statin/cholesterol/mevalonate pathway terms. Additional subtype keywords were expanded selectively to optimize sensitivity where variant labels may appear without the word ‘porokeratosis’ or are inconsistently indexed (e.g. ‘Mibelli’); PP/PPPD were not included as standalone abbreviations because the full entity names include ‘porokeratosis’ (and are therefore captured by the parent term), while the abbreviations are nonstandard and potentially ambiguous.

PubMed (no filters):

- (‘porokeratosis’[Title/Abstract] OR ‘disseminated superficial actinic porokeratosis’[Title/Abstract] OR ‘linear porokeratosis’[Title/Abstract] OR ‘porokeratosis of Mibelli’[Title/Abstract] OR ‘Mibelli’[Title/Abstract] OR ‘porokeratosis ptychotropica’[Title/Abstract])
- AND (‘statin’[Title/Abstract] OR ‘simvastatin’[Title/Abstract] OR ‘lovastatin’[Title/Abstract] OR ‘atorvastatin’[Title/Abstract] OR ‘cholesterol’[Title/Abstract] OR ‘mevalonate pathway’[Title/Abstract])

Scopus (no filters):

- (TITLE-ABS-KEY(porokeratos*) OR TITLE-ABS-KEY(‘disseminated superficial actinic porokeratosis’ OR DSAP OR ‘porokeratosis ptychotropica’ OR ‘porokeratosis of mibelli’ OR ‘linear porokeratosis’ OR ‘facial porokeratosis’ OR ‘solar facial porokeratosis’))
- AND
- (TITLE-ABS-KEY(statin* OR simvastatin OR lovastatin OR atorvastatin OR pravastatin OR rosuvastatin OR ‘HMG-CoA reductase’ OR mevalonat* OR ‘topical statin*’ OR ‘statin cream’ OR ‘statin ointment’))
- OR TITLE-ABS-KEY(‘cholesterol/lovastatin’ OR ‘lovastatin/cholesterol’ OR ‘simvastatin cholesterol’ OR (cholesterol W/3 (statin* OR cream OR ointment OR therapy)))

Embase via Ovid (filters: English, human):

- (porokeratosis OR ‘disseminated superficial actinic porokeratosis’ OR ‘linear porokeratosis’ OR ‘porokeratosis of mibelli’ OR mibelli OR ‘porokeratosis ptychotropica’)
- AND (statin OR simvastatin OR lovastatin OR atorvastatin OR cholesterol OR ‘mevalonate pathway’)

We also searched ClinicalTrials.gov (register) using the Expert Search fields Condition/Disease = ‘Porokeratosis’ and Other terms = ‘Statin’; no status or location filters; last searched 8 December 2025. The search returned one record (NCT04359823) with already-published results. No automation tools were used.

### Eligibility criteria

#### Inclusion

Clinical studies (trials and case series/reports) of patients with porokeratosis treated with topical statins (alone or with cholesterol/other agents) that reported patient-level clinical outcomes were eligible (English language). Given the recognized scarcity of high-level evidence in porokeratosis, our objective was to compile the most comprehensive, contemporary mapping of clinical experience. We therefore adopted broad inclusion criteria to capture any source with unique patient-level outcomes, including two conference abstracts and one patient viewpoint with photo-documented outcomes, in addition to peer-reviewed full reports. To maintain methodological rigour, these low-certainty sources were appraised with Joanna Briggs Institute (JBI) checklists and handled conservatively: abstracts were used only for descriptive counts, with the aggregate-only abstract additionally contributing to a prespecified sensitivity analysis of aggregate-only data; the patient viewpoint was treated as a single case report and included in patient-level tallies. None of these sources informed randomized GRADE comparisons.

#### Exclusion

Nonclinical articles (e.g. reviews, commentaries, editorials and *in vitro*/animal studies) and reports without patient-level outcomes were excluded.

### Study selection and data extraction

Titles/abstracts and full texts were screened against eligibility criteria by two independent reviewers; disagreements were resolved by discussion/consensus. Two reviewers performed data extraction with a piloted sheet and two additional authors verified all entries, resolving discrepancies by consensus. Extracted items included study design, porokeratosis subtype, intervention (statin and adjuncts), regimen, duration/follow-up, outcomes and adverse events. Authors were not contacted for missing data; no imputation was performed. When two or more reports described the same patient, we collated them and treated them as one patient or study; the later or more complete report resolved discrepancies.

**Figure 1:**
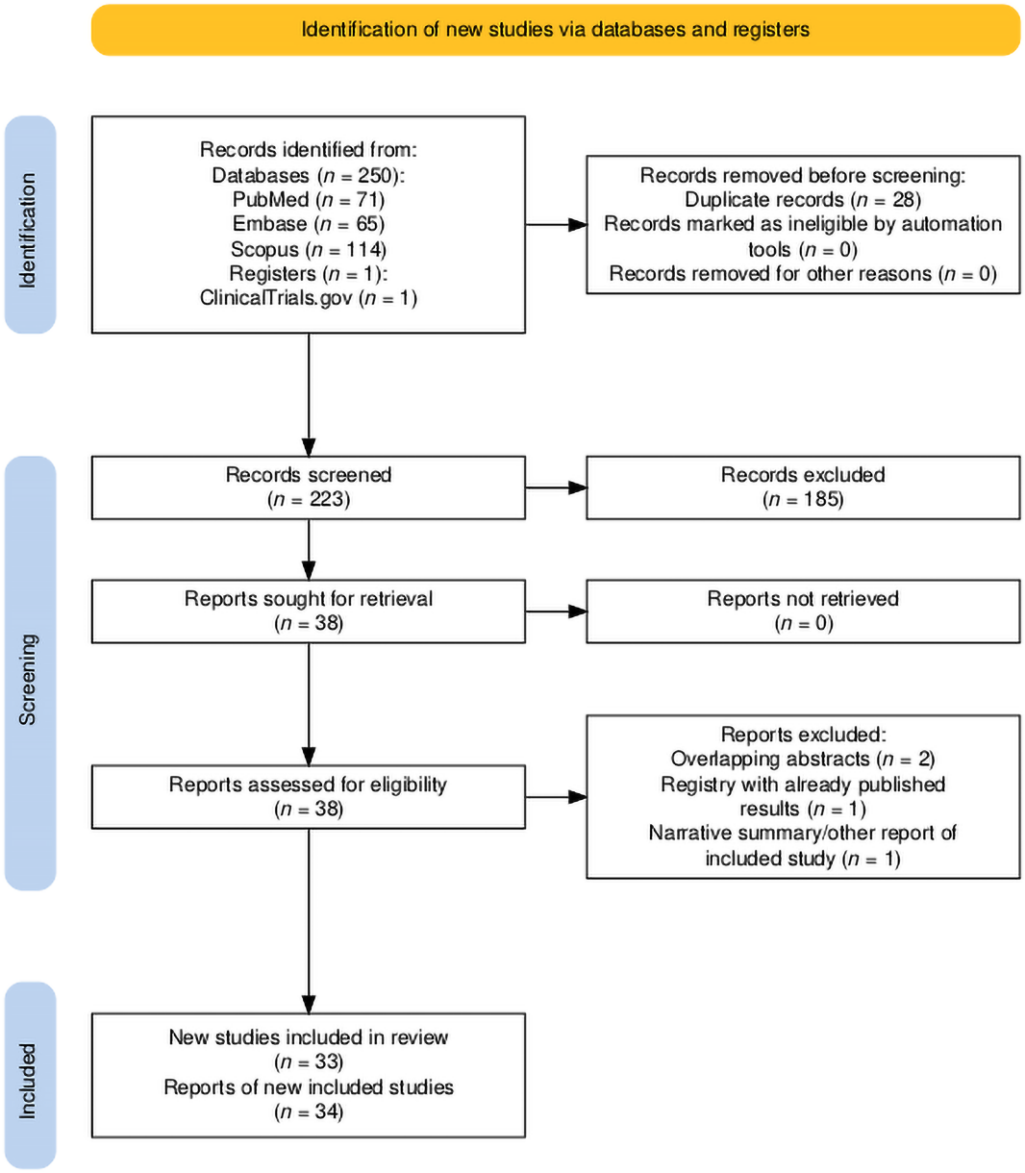
PRISMA 2020 flow diagram of the study selection. PubMed, Embase and Scopus were searched from inception through 8 December 2025. ClinicalTrials.gov was searched on 8 December 2025. One ClinicalTrials.gov record that was also indexed in Embase was counted once under ‘Register’ and excluded from inclusion because its results were published in a peer-reviewed article. Two sequential reports describing the same patient were merged and counted as one study. All counts in the diagram reflect de-duplication across sources. In the ‘Records excluded’ box, ‘Overlapping abstracts’ denotes conference abstracts reporting data already published in full; ‘Narrative summary/other report’ denotes a nonprimary report (e.g. narrative summary) related to an included study.

From 250 database records and 1 register record, 28 duplicates were removed, leaving 223 unique records screened at title and abstract level. Thirty-eight full-text reports were assessed for eligibility. Four were excluded (two overlapping abstracts; one register record with already-published results; one narrative summary/other report of an included study). Two sequential reports described the same patient and were merged at extraction, yielding 33 included studies (34 reports) (Figure 1).

### Outcome classification

To enable cross-study synthesis, author language was mapped to prespecified categories: complete/near-complete response (explicit clearance/near-clearance), partial response (‘marked,’ ‘significant’ or ‘moderate’ improvement indicating substantial but incomplete resolution) and poor/no response (minimal change/none). Recurrence (reappearance of lesions after response) and adverse events were recorded as reported. When multiple timepoints were available, the final on-treatment assessment (or the longest follow-up if post-treatment only) was used.

### Data synthesis, effect measures and risk-of-bias assessment

Given heterogeneity in designs and outcomes, a narrative synthesis was performed and structured by study type (randomized trials, case series, case reports, abstracts, patient viewpoint) and porokeratosis subtype where feasible. Effect measures were descriptive only: we summarized simple proportions of patients achieving each prespecified response category across studies; no statistical pooling or comparative modelling was attempted. We planned subgroup syntheses by study design (randomized vs. nonrandomized) and porokeratosis subtype [DSAP, LP, porokeratosis ptychotropica (PPt), porokeratosis of Mibelli (PM), punctate porokeratosis/porokeratosis palmaris et plantaris disseminata (PP/PPPD), solar facial porokeratosis (SFP)], with descriptive stratification by vehicle/adjuncts (ointment/cream/gel, laser-­assisted delivery) where feasible. Sensitivity analyses were prespecified to (i) include aggregate-only case series that did not report per-patient response tiers and (ii) test classification assumptions (e.g. handling of ‘minor improvement’ and multi-timepoint reports). Because a minority of reports used adjunctive therapies (e.g. fractional CO₂ pretreatment and short acitretin exposure), we planned a sensitivity analysis excluding such cases to gauge confounding. Nonstatin topicals used before statin initiation (e.g. a keratolytic trial in one case of PP) were recorded but not classified as adjuncts.

Risk of bias for randomized trials used Cochrane Risk of Bias 2 (RoB 2) (Table S1; see Supporting Information). All nonrandomized evidence (case series and case reports, including one aggregate-only series and one patient viewpoint report) was appraised with the relevant JBI checklists; item-level judgements are shown in Table S2 (see Supporting Information), with several items marked ‘unclear/not applicable’ where details were insufficient.

### Reporting bias and certainty of evidence

We did not apply formal small-study or publication/reporting bias methods (e.g. funnel plots and Egger tests) because we conducted no quantitative meta-analyses and each synthesis contained too few comparable studies (randomized evidence comprised three trials; nonrandomized evidence consisted of single-arm case series or case reports with heterogeneous, nonstandardized outcomes). Under these conditions such methods are not applicable. Protocol registration and prespecified outcomes (PROSPERO) were used to mitigate selective reporting. We applied GRADE only to comparative questions addressed by randomized evidence: (i) topical statin vs. control for DSAP severity; (ii) statin + cholesterol vs. statin alone; and (iii) serious adverse events. Certainty was judged across risk of bias, inconsistency, indirectness, imprecision and publication bias. Single-arm case series or case reports were not graded; for these, we provide narrative certainty statements aligned with GRADE concepts. We did not construct a formal Summary-of-Findings (SoF) table because we performed no quantitative pooling and only three small, heterogeneous randomized trials contributed to a limited set of comparative questions; presenting tabulated effect estimates in SoF format could imply spurious precision and comparability. Abstract-only and patient viewpoint items were treated as low-­certainty evidence and did not inform randomized GRADE judgements.

### Deviations from protocol

Given the scarcity of data in this area, we broadened our plan before extracting from the affected sources to include two conference abstracts and one patient viewpoint with photo-­documented outcomes. Abstracts were used only for descriptive tallies; the aggregate-only abstract additionally informed a prespecified sensitivity analysis of aggregate-only data. The patient viewpoint was treated as a single case report and included in patient-level counts. All three sources were appraised with JBI checklists and were excluded from randomized GRADE comparisons.

## Results

### Study yield and designs

We included 33 studies (2020–25): 3 randomized trials [1 parallel-group DSAP randomized controlled trial (RCT) and 2 randomized split-body trials],^13–15^ 9 case series including 2 abstract-only reports, 20 case reports and 1 patient viewpoint [Table 1; Table S3 (see Supporting Information)].^16–46^ One sequential two-report case of LP was counted once at the patient level to avoid duplication.^16,17^ Overall, 162 patients were analysed.

**Table 1:** Randomized trials (parallel-group and split-body): design and primary signal

Study	No. of patients studied	Subtype(s)	Regimen	Outcome	Duration	AEs
Santa Lucia 2023, parallel-group RCT^13^	31 randomized; 24 analysed for primary outcome	DSAP	2% lovastatin vs. 2% lovastatin + 2% cholesterol cream, once or twice daily	DSAP-GASI decreased similarly in both arms; no between-arm difference (*P* ≈ 0.98)	12 weeks (3 months)	Mild myalgia (*n* = 2), CK elevation (*n* = 1), application discomfort/rash (*n* = 5); all resolved
Chen 2025, randomized, single-blind, split-body RCT^14^	16	PPt	2% simvastatin cream vs. 2% simvastatin + 2% cholesterol cream, twice daily	PPASI and pruritus improved in both arms; no between-arm difference at final assessment	48 weeks total (initial 8-week blinded phase + 40-week extension)	Mild transient pruritus/rash in ∼10 participants; resolved; no serious AEs
Byth 2021, open-­label, laterality-­randomized, split-body (within-patient) clinical trial^15^	8	DSAP	2% simvastatin + 2% cholesterol cream vs. contralateral vehicle	Treated limbs improved vs. control across lesion count, erythema, scale, pruritus and patient global; reported ORs ≈0.12–0.33 favouring active	6 weeks	Local irritation in 3 patients; 1 discontinued but included in ITT; no systemic AEs

Parallel-group randomized controlled trial (RCT) denotes between-patient allocation; split-body denotes within-patient side allocation. For split-body trials, randomization refers to side allocation. AEs, adverse events; CK, creatine kinase; DSAP, disseminated superficial actinic porokeratosis; DSAP-GASI, DSAP Global Assessment of Severity Index; ITT, intention-to-treat; OR, odds ratio; PPt, porokeratosis ptychotropica; PPASI, Porokeratosis Ptychotropica Area and Severity Index.

### Porokeratosis subtypes

DSAP constituted most cases (*n* = 122/162; 75.3%), followed by PPt (*n* = 19/162; 11.7%) and LP (*n* = 15/162; 9.3%); less common subtypes included PM (*n* = 2/162; 1.2%), PPPD (*n* = 2/162; 1.2%), PP (*n* = 1/162; 0.6%) and SFP (*n* = 1/162; 0.6%).

### Regimens and duration

Most preparations were lovastatin or simvastatin 1–2%, delivered either as cream (*n* = 15/33 studies, including all three randomized trials) or ointment (*n* = 15/33); gel and lotion each appeared once, and one DSAP series used mixed or unspecified vehicles (Table 1; Table S3). Treatment duration varied by design. Randomized trials evaluated primary endpoints after 6–12 weeks (with one PPt trial including a masked 8-week phase followed by a 40-week extension).^13–15^ In nonrandomized reports, many LP and localized cases were assessed after approximately 2–3 months of therapy,^18–21^ whereas DSAP case series and long-term case reports frequently continued topical statins for 6–24 months, with median or last follow-up around 12–23 months and a maximum DSAP follow-up of 46 months in a 17-patient lovastatin or cholesterol series.^22–29^ The longest follow-up in PPt was 24–26 months in a two-patient simvastatin/cholesterol ointment series.^30^

### Risk of bias and certainty

Across the three randomized datasets, risk of bias ranged from some concern to high. The DSAP parallel-group RCT was patient-blinded but analysed 24 of 31 randomized participants for the ­primary endpoint without intention-to-treat (ITT) sensitivity analyses, yielding high risk of bias from missing outcome data.^13^ The DSAP open-label, laterality-randomized, split-body (within patient) trial used computer-generated random allocation of sides and ITT with complete 6-week outcomes;^15^ however, because outcomes were unblinded, subjective ordinal scales, the risk of bias from outcome measurement was high, with some concerns about allocation concealment. The PPt randomized, single-blind, split-body trial included a masked 8-week treatment phase with ITT analysis.^14^ Allocation was described as by envelopes, but their adequacy was not reported, so the overall risk of bias was judged as having some concerns. None of the trials was unequivocally low risk across all RoB 2 domains. Trial-level RoB 2 findings are summarized in the text; item-level RoB 2 judgements are shown in Table S1. Nonrando­mized evidence (case series and case reports) was generally of low certainty owing to uncontrolled designs and heterogeneity. JBI appraisals for all non randomized studies (case series and case reports) are provided in Table S2. On GRADE assessment, certainty was low that topical statins improve DSAP severity and moderate that cholesterol add-on confers no additional benefit; serious adverse events were low. For other porokeratosis subtypes, evidence was limited to small, uncontrolled case series and case reports, and certainty was rated very low. No formal small-study or publication-bias tests were applicable; qualitatively, reporting bias is probable given the predominance of case reports and case series. The two abstract-only series and the single patient viewpoint were JBI-appraised and used only for descriptive counts (with the aggregate-­only abstracts also in a prespecified sensitivity analysis) and did not inform randomized GRADE judgements.

### Outcome harmonization

We prespecified responder categories (complete/near-complete, partial, poor/no response) and applied them to studies with patient-level data. In 98 classifiable patients from nonrandomized reports, complete/near complete responses occurred in 16/98 (16%), partial responses in 67/98 (68%) and poor/no responses in 15/98 (15%) (Table 2). The majority of nonrandomized patients had DSAP (*n* = 76/98; 78%), with complete/near-complete responses in 15 of 76 (20%) and partial responses in 53 of 76 (70%). As author-level data in one LP series emphasized ‘no significant improvement in most’, those five patients with LP were conservatively classified as having a poor/no response;^31^ a sensitivity analysis treating ‘minor improvement’ as partial did not alter conclusions. These patient-level tallies are shown in Table 2.

**Table 2:** Response by subtype (nonrandomized; patient-level; primary analysis).

Subtype	*n*	Complete/near-complete response *n*	Partial response	Poor/no response
Disseminated superficial actinic porokeratosis	76	15 (20)	53 (70)	8 (11)
Linear porokeratosis	14	0 (0)	8 (57)	6 (43)
Porokeratosis ptychotropica	3	0 (0)	2 (67)	1 (33)
Porokeratosis palmaris et plantaris disseminata	2	0 (0)	2 (100)	0 (0)
Punctate porokeratosis	1	0 (0)	1 (100)	0 (0)
Porokeratosis of Mibelli	1	1 (100)	0 (0)	0 (0)
Solar facial porokeratosis	1	0 (0)	1 (100)	0 (0)
Total	98	16 (16)^a^	67 (68)	15 (15)^b^

Data are presented as n (%). Counts include only reports with per-patient outcomes. Randomized trials and aggregate-only series were excluded; see the ‘Sensitivity analyses’ section for the definition and sensitivity totals.

^a^Complete responses.

^b^Poor responses.

### Efficacy by design and subtype

#### Randomized evidence: three trials

A parallel-group RCT involving patients with DSAP (*n* = 31 randomized; 24 analysed for the primary endpoint)^13^ showed improvement in Disseminated Superficial Actinic Porokeratosis–Global Assessment of Severity Index (DSAP-GASI) in both arms, with no between-arm difference. An open-label, laterality-­randomized, split-body (within-patient) trial involving patients with DSAP (*n* = 8)^15^ found the active side improved vs. contralateral control across lesion count, erythema, scale, pruritus and patient global (reported odds ratios ∼0.12–0.33 favouring active). A randomized, single-blind, split-body trial involving patients with PPt (*n* = 16)^14^ showed improvement in both arms (simvastatin vs. simvastatin + cholesterol) during the 8-week blinded phase; there was no between-arm difference at the final assessment after the extension. Study-level details are summarized in Table 1.

##### Certainty (GRADE)

DSAP improvement vs. control: low (downgraded for risk of bias and imprecision); cholesterol add-on vs. statin alone: moderate (concordant null across two randomized datasets with some concerns for bias); serious adverse events: low (low patient numbers; sparse events); other subtypes: very low (certainty downgraded for indirectness and heterogeneity).

#### Uncontrolled evidence in disseminated superficial actinic porokeratosis

Nonrandomized DSAP data comprised three cohorts of 17–20 patients each,^23,27,28^ together with smaller series and case reports discussing statin monotherapy or statin–cholesterol combinations.^23,27,28^ Across these studies, outcomes mirror the pooled DSAP estimates in Table 2 (≈20% complete or near-complete clearance, ≈70% partial improvement, ≈10% poor or no response), indicating that DSAP is the best-characterized porokeratosis subtype but that most patients achieve only partial responses and maintenance approaches remain heterogeneous.

#### Linear porokeratosis and other subtypes

LP responses were variable and appeared to be thickness- dependent. In a sequential single-patient, bilateral LP comparison, lovastatin + cholesterol showed no advantage over statin monotherapy;^16,17^ across the remaining LP reports, most were partial with several poor/no responses (Table 2). The single patient with PP had prior nightly topical salicylic acid, which was discontinued before starting lovastatin/cholesterol, and the patient subsequently achieved a partial response.^32^ For PM, one patient-level case achieved near-complete clinical flattening by day 40 on lovastatin/cholesterol (a second PM appears only in an aggregate-level series).^24^,^33^ There was one patient-level case of SFP with a partial response.^34^

#### Time to response, durability and maintenance

The onset of visible improvement typically occurred within 2–12 weeks across designs and subtypes.^33,35–38^ Relapse was described after treatment interruption or on reduced-frequency maintenance in some reports; maintenance preserved gains when used.^33,38^

#### Safety

Topical statins were generally well-tolerated. Local irritation or pruritus occurred infrequently. Allergic contact dermatitis (ACD) to simvastatin was confirmed by patch testing in at least three patients; discontinuation resolved symptoms.^20,22,28^ In the DSAP RCT, transient myalgia (*n* = 2) and creatine kinase (CK; *n* = 1) were reported without sequelae.^13^ No serious treatment-related adverse events were identified across randomized studies.^13–15^

##### Certainty (GRADE)

Low for the absence of serious treatment-related adverse events (small total number of patients; sparse harms reporting).

#### Cholesterol add-on

Across randomized evidence in DSAP and PPt, adding cholesterol to a statin did not confer incremental benefit over statin monotherapy; improvements were observed in both arms.^13,14^ Two ­separate RCTs (one in DSAP, one in PPt) found no significant difference in outcomes between statin monotherapy and statin–­cholesterol combination therapy.^13,14^ Case-level signals were mixed and hypothesis-generating (e.g. one SFP split-face with numerically similar scores and one cholesterol-only improvement).^34^ We therefore interpret cholesterol as optional, not required.

##### Certainty (GRADE)

Moderate that adding cholesterol confers no incremental benefit (concordant null across two randomized datasets, with some concerns for bias).

### Sensitivity analyses

#### Aggregate only sensitivity

To test robustness, we repeated the response tally after adding the only aggregate-only series by Herrero-Ruiz *et al.* (*n* = 9; mixed DSAP/LP/PM; simvastatin 2% cream applied once daily), which reported improvement in eight of nine patients without per-­patient tiers.^24^ We therefore counted these as eight partial and one poor response. Totals shifted from 16 of 98 (16%) complete/near-complete responses, 67 of 98 (68%) partial responses and 15 of 98 (15%) poor/no responses, to 16 of 107 (15.0%) complete/near-complete responses, 75 of 107 (70.1%) partial responses and 16 of 107 (15.0%) poor/no responses. The direction and conclusions were unchanged. Aggregate-only refers to reports lacking per-patient response categories; randomized trials were intentionally excluded from this analysis.

#### Adjuncts sensitivity

Excluding the two patients with DSAP who received adjuncts (fractional CO_2_ pretreatment and short-course acitretin) did not materially change response distributions: totals shifted from 16 of 98 (16%) complete/near-complete responses, 67 of 98 (68%) partial responses and 15 of 98 (15%) poor responses to 16 of 96 (17%) complete/near-complete responses, 65 of 96 (68%) partial responses and 15 of 96 (16%) poor responses. Conclusions were unchanged.

## Discussion

Across 33 studies (162 patients), topical statins improved poro­keratosis severity with a predominance of partial responses; ­complete/near-complete clearances occurred in a minority of patient-level reports [primary analysis: complete/near-complete 16% (*n* = 16/98), partial 68% (*n* = 67/98), poor/no response 15% (*n* = 15/98)]. In DSAP, randomized evidence showed improvement on validated indices, and adding cholesterol did not confer incremental benefit over statin alone in the parallel DSAP RCT and the randomized split-body PPt trial.^13,14^ LP responses were variable and appeared to be thickness-dependent.^31,39^ Limited PM evidence includes one patient-level case with a near-complete response and one additional case of PM embedded in an aggregate-only series (per-patient tier unavailable);^24,33^ PP and SFP each have single cases of partial response.^32,34^ Onset typically occurred within weeks, maintenance prevented relapse in some reports.^33,38^ Safety was favourable overall; patch test-confirmed simvastatin ACD has been reported in at least three patients in nonrandomized evidence and clinical series,^20,22,28^ and in the DSAP RCT there were cases of transient myalgia (*n* = 2) and one CK elevation, all resolved without sequelae; no serious treatment-related systemic AEs were identified.^13^

Based on this synthesis of limited evidence, clinicians managing symptomatic or cosmetically significant DSAP may consider a trial of topical statin monotherapy (e.g. lovastatin or simvastatin 1–2% in an ointment or cream base). An initial trial of 8–12 weeks appears reasonable as a starting point. Our findings do not support the routine addition of cholesterol.^13–15^ Set expectations for partial improvement; anticipate attenuated responses in thick/long-standing LP plaques;^31,39^ and consider maintenance dosing to reduce relapse.^33^ If dermatitis develops, treatment cessation and patch testing should be considered; patch test-­confirmed simvastatin ACD has been reported in at least three patients.^20,22,28^ Routine laboratory monitoring is not required; targeted testing may be considered if patients report systemic symptoms.^13^ Compounding costs can limit access in some settings (Box 1).^25^ Whether partial clinical improvement with topical statins translates into reduced keratinocyte cancer risk in DSAP or LP remains unknown; none of the included studies was designed or powered to evaluate malignant transformation.^2–4^

Box 1 **Practice points for topical statins in porokeratosis**

**Table:** 

**Start:** Consider prescribing lovastatin or simvastatin 1–2% in an ointment or cream base, applied once or twice daily for an 8–12-week initial trial. Reassess for response. Do not routinely add cholesterol, as evidence does not support its superiority.^13–15^
**Expect:** Counsel patients that partial improvement is the most common outcome. Complete or near-complete responses occur in a minority (∼16%). Thicker plaques, such as in linear porokeratosis, are less likely to respond well.^31,39^ **Maintain:** For patients who respond, consider continuing therapy at the lowest effective frequency (e.g. several times weekly) to maintain improvement. Relapse is common after discontinuation.^33^ **Adjuncts (selective):** For refractory, hyperkeratotic lesions consider adding a keratolytic agent (e.g. salicylic acid)^32^ or discussing procedural adjuncts like fractional CO_2_ laser to enhance penetration,^40^ although evidence is preliminary and based on case reports. **Monitor:** Advise patients to watch for signs of dermatitis. If it develops, discontinue therapy and consider patch testing, as allergic contact dermatitis to simvastatin has been reported. Routine systemic laboratory monitoring is not necessary unless the patient reports systemic symptoms such as myalgia.^20,22^ **Access:** Be aware that these are compounded, off-label preparations. Cost and availability may be significant barriers for patients.^25^ **Strength of evidence:** Low for disseminated superficial actinic porokeratosis improvement with topical statins; moderate against routine cholesterol add-on (no consistent additional benefit); low for safety (serious adverse events rare but underpowered); very low for other porokeratosis subtypes.

Therapeutic benefit is biologically plausible via local HMG-CoA reductase inhibition in mevalonate pathway-mediated dyskeratosis;^9–12^ mechanistic inferences should be interpreted cautiously given the small, heterogeneous trial base.

Strengths include a comprehensive search, prespecified harmonization of outcomes and subtype-level synthesis. Limitations mirror the field: small trials, short follow-up, heterogeneity in drug/vehicle/dose, qualitative outcome wording in many reports and predominance of nonrandomized designs; a few reports used adjuncts (fractional CO₂; brief acitretin), limiting attribution in those cases, although sensitivity analyses excluding them did not alter conclusions. Critically, the DSAP parallel RCT analysed 24 of 31 randomized patients without ITT sensitivity analyses (high risk of bias due to missing outcome data)^13^ and the DSAP split-body trial was open-label with unclear concealment (some concerns).^15^ Reporting bias is probable in this field: positive or partial responses, especially in case reports and case series, are more often reported than null findings. We included two abstract-only series and one patient viewpoint to map all patient-level experience; these were JBI-appraised, used only for descriptive counts (with the aggregate-only abstract also used in a prespecified ­sensitivity analysis) and did not inform randomized GRADE judgements. This potential bias may inflate response rates and contributes to our low or very-low certainty ratings. Transparent, ­item-level risk-of-bias and appraisal is provided (RoB 2 in Table S1; JBI in Table S2). We searched trial registries for additional information; we did not contact study authors for the abstract-only and viewpoint items, which may leave residual uncertainty for those reports.

Despite these inherent limitations, which reflect the state of the field, this review’s strength lies in its comprehensive and methodologically transparent synthesis, providing the clearest possible picture of the current evidence base and a solid foundation for future investigation.

Research priorities should include:

- Head-to-head DSAP RCTs: statin vs. statin + cholesterol with identical vehicles, validated indices (DSAP-GASI), independent photo-adjudication and patient-important outcomes.
- Maintenance trials: tapering schedules and relapse kinetics after response.
- Penetration strategies for LP: keratolysis and procedural delivery (e.g. fractional CO_2_) tested prospectively in thick plaques.
- Safety pharmacovigilance: molecule/vehicle-specific risk of ACD and pragmatic management algorithms.
- Oncological outcomes: prospective cohorts/registries with long-term follow-up to evaluate malignant transformation and keratinocyte cancer outcomes in treated vs. untreated poro­keratosis, as current studies were not designed or powered for this endpoint.

Topical statins are a mechanism-based, nondestructive option for porokeratosis, showing a dominant partial-improvement profile, modest complete-clearance rates and a favourable tolerability signal. The evidence is strongest for DSAP, where statin monotherapy can be considered a therapeutic option, potentially offering partial improvement with a favourable safety profile. Certainty remains low overall, and evidence for non-DSAP variants is very limited; therefore, treatment should be individualized while larger, standardized trials define optimal formulations and clarify the role, if any, of cholesterol.

## Supplementary Material

vzag043_Supplementary_Data

## Data Availability

All data are included in the article and its Supporting Information. Screening logs, extraction sheets and risk-of-bias forms are available from the corresponding author on reasonable request.
