# Supplementary material for: Efficacy and safety of topical statins for porokeratosis: a systematic review and practice-guided synthesis
Source: Skin Health Dis. 2026 Jun 9;6(4):383–92. doi: 10.1093/skinhd/vzag043 (PMC13424892; doi:10.1093/skinhd/vzag043)
Supplement: vzag043_Supplementary_Data [file vzag043_supplementary_data.zip › Table S1.docx]

**Table S1. Risk of Bias (RoB 2) for Randomized Trials**

| **Trial** | **Subtype (s); n** | **Design** | **D1 Randomization process** | **D2 Deviations from intended interventions (effect of assignment)** | **D3 Missing outcome data** | **D4 Measurement of the outcome** | **D5 Selection of the reported result** | **Overall RoB** |
| --- | --- | --- | --- | --- | --- | --- | --- | --- |
| Santa Lucia 2023 (13) | DSAP; 31 (24 analyzed for the primary endpoint) | Parallel-group RCT | Low (computer-generated blocks; allocation concealed; patient-blinded) | Some concerns (no ITT; 24/31 analyzed) | High (24/31 analyzed; no sensitivity analyses) | Low (assessor-blinded; standardized photo scoring) | Low (protocol/SAP provided; prespecified outcomes) | High (driven by D3) |
| Byth 2021 (15) | DSAP; 8 | Open-label, randomized, split-body (within patient) trial | Some concerns (side allocation randomized; concealment not described) | Low (ITT with complete 6-week outcomes) | Low (complete at 6 weeks) | High (unblinded, subjective ordinal scales) | Some concerns (no protocol/SAP) | High (driven by D4) |
| Chen Z 2025 (14) | PPt; 16 | Randomized split-body RCT (8-week masked phase) | Some concerns (randomized side assignment; allocation concealed via envelopes; adequacy of concealment not reported) | Some concerns (per-protocol analysis; not ITT) | Low (no missing blinded-phase outcomes) | Low (masked outcome assessment during 8-week phase) | Some concerns (no documented SAP) | Some concerns |

RoB 2 domains: D1 randomization process; D2 deviations from intended interventions (effect of assignment to intervention); D3 missing outcome data; D4 measurement of the outcome; D5 selection of the reported result. Judgments follow RoB 2 guidance (Low / Some concerns / High). Overall risk is High if any domain is High. Abbreviations: n =patients; ITT= intention-to-treat; SAP= statistical analysis plan; PPt= porokeratosis ptychotropica; DSAP= disseminated superficial actinic porokeratosis. In split-body trials, randomization refers to side allocation.
