# Supplementary material for: Efficacy and safety of topical statins for porokeratosis: a systematic review and practice-guided synthesis
Source: Skin Health Dis. 2026 Jun 9;6(4):383–92. doi: 10.1093/skinhd/vzag043 (PMC13424892; doi:10.1093/skinhd/vzag043)
Supplement: vzag043_Supplementary_Data [file vzag043_supplementary_data.zip › Table S2.docx]

**Table S2. JBI Critical Appraisal — Nonrandomized Evidence**

Part A: Case Series (JBI 10‑item checklist); Part B: Case Reports (JBI 8‑item checklist).

**Part A. Case Series (JBI 10‑item checklist)**

| **Study** | **Subtype(s); n** | **Q1** | **Q2** | **Q3** | **Q4** | **Q5** | **Q6** | **Q7** | **Q8** | **Q9** | **Q10** | **Overall appraisal** |
| --- | --- | --- | --- | --- | --- | --- | --- | --- | --- | --- | --- | --- |
| Tomsitz 2022 (23) | DSAP; 7 | Y | Y | Y | U | U | Y | Y | Y | Y | NA | Include |
| Albanell-Fernández 2023 (18) | PPt; 2 | N | Y | Y | U | U | Y | Y | Y | Y | NA | Include |
| Atzmony 2020 (16) | DSAP (=1), PPPD (=2), LP (=2); 5 | Y | Y | Y | U | U | Y | Y | Y | Y | NA | Include |
| Barrabès-Torrella 2024 (30) | DSAP; 20 | U | U | U | U | U | N | U | Y | U | NA | Include |
| Herrero-Ruiz 2024 (31) | DSAP (n=7), LP (n=1), PM (n=1) | U | Y | U | U | U | Y | U | Y | U | NA | Include |
| Jerjen 2020 (25) | DSAP; 2 | N | Y | U | U | U | Y | Y | Y | Y | NA | Include |
| Saleva‐Stateva 2020 (29) | LP; 5 | N | Y | Y | U | U | Y | Y | Y | Y | NA | Include |
| Tan 2024  (43) | DSAP; 17 | Y | Y | Y | Y | Y | Y | Y | Y | Y | Y | Include |
| Lang 2025 (44) | DSAP;19 | Y | Y | Y | Y | Y | Y | Y | Y | Y | Y | Include |

Coding: Y = yes; N = no; U = unclear; NA = not applicable. JBI Case Series items: Q1 inclusion criteria; Q2 standard/reliable measurement; Q3 valid identification; Q4 consecutive inclusion; Q5 complete inclusion; Q6 demographics; Q7 clinical information; Q8 outcomes/follow‑up; Q9 site/clinic details; Q10 statistical analysis. For case series, Q10 (statistical analysis) is often NA unless formal statistical analysis is reported. Appraisals reflect reporting quality and do not imply exclusion; all studies were included in the synthesis. Study labels show first author and year; numbers in parentheses map to the main Reference list. For case series, Q10 (statistical analysis) is typically NA.

**Part B. Case Reports (JBI 8‑item checklist)**

| **Study** | **Subtype** | **Q1** | **Q2** | **Q3** | **Q4** | **Q5** | **Q6** | **Q7** | **Q8** | **Overall appraisal** |
| --- | --- | --- | --- | --- | --- | --- | --- | --- | --- | --- |
| Aerts 2023 (28) | DSAP | Y | Y | Y | Y | Y | Y | Y | Y | Include |
| Alakeel 2023 (22) | LP | Y | Y | Y | Y | Y | Y | Y | Y | Include |
| Badea 2024 (36) | PPt | Y | Y | Y | Y | Y | Y | Y | Y | Include |
| Blue 2021 (19) | LP | Y | Y | Y | Y | Y | Y | Y | Y | Include |
| Buhle 2022 (17) | LP | Y | Y | Y | Y | Y | Y | Y | Y | Include |
| Cabo 2024 (39) | DSAP | Y | Y | Y | Y | Y | Y | U | Y | Include |
| Chen K 2025 (27) | LP | Y | Y | Y | Y | Y | Y | Y | Y | Include |
| Diep 2022 & 2023 (41,42) | LP | Y | Y | Y | Y | Y | Y | Y | Y | Include |
| Gao 2025 (37) | DSAP | U | Y | Y | Y | Y | Y | Y | Y | Include |
| Maredia 2024 (20) | LP | Y | Y | Y | Y | Y | Y | Y | Y | Include |
| Maronese 2021 (24) | DSAP | Y | Y | Y | Y | Y | Y | Y | Y | Include |
| McFeely 2021 ‡ (34) | DSAP | N | U | U | N | Y | Y | N | Y | Include |
| Nguyen 2024 (21) | PM | Y | Y | Y | Y | Y | Y | Y | Y | Include |
| Peng 2024 (26) | SFP | Y | Y | Y | Y | Y | Y | Y | Y | Include |
| Janaani 2024 (38) | DSAP | Y | Y | Y | Y | Y | Y | Y | Y | Include |
| Raison-Peyron 2025 (35) | LP | Y | Y | Y | Y | Y | Y | Y | Y | Include |
| Ryan 2023 (33) | PP | Y | Y | Y | Y | Y | Y | Y | Y | Include |
| Sultan 2023 (40) | DSAP | Y | Y | Y | Y | Y | Y | Y | Y | Include |
| Ugwu 2020 (32) | DSAP | Y | Y | Y | Y | Y | Y | Y | Y | Include |
| Howe 2025 (45) | DSAP | Y | Y | Y | Y | Y | Y | Y | Y | Include |
| Möller 2025 (46) | DSAP | Y | Y | Y | Y | Y | Y | Y | Y | Include |

‡ McFeely 2021 is a patient-viewpoint item; appraised against JBI case-report domains for completeness, retained in synthesis.

Coding: Y = yes; N = no; U = unclear; NA = not applicable. JBI Case Report items: Q1 demographics; Q2 history/timeline; Q3 clinical condition; Q4 diagnostic tests/assessment; Q5 intervention; Q6 post‑intervention condition; Q7 adverse/unanticipated events; Q8 takeaway lessons. Study labels list first author and year; numbers in parentheses map to the main Reference list. No separate supplementary reference list is provided. Appraisals reflect reporting quality and do not imply exclusion; all case reports were included in the synthesis.

Abbreviations (apply to S2A-B): DSAP = disseminated superficial actinic porokeratosis; JBI = Joanna Briggs Institute; LP = linear porokeratosis; PPt = porokeratosis ptychotropica; PP = punctate porokeratosis; PPPD = porokeratosis plantaris, palmaris et disseminata; PM = porokeratosis of Mibelli; SFP = solar facial porokeratosis.
