# Supplementary material for: Efficacy and safety of topical statins for porokeratosis: a systematic review and practice-guided synthesis
Source: Skin Health Dis. 2026 Jun 9;6(4):383–92. doi: 10.1093/skinhd/vzag043 (PMC13424892; doi:10.1093/skinhd/vzag043)
Supplement: vzag043_Supplementary_Data [file vzag043_supplementary_data.zip › Table S3.docx]

**Table S3. Detailed characteristics of all non-randomized reports (case series, case reports, abstracts, viewpoint)**

| **Study** | **N** | **Subtype(s)** | **Regimen** | **Outcome** | **Duration** | **Adverse events (AEs)** |
| --- | --- | --- | --- | --- | --- | --- |
| Atzmony 2020 (16) | 5 | DSAP (=1), PPPD (=2), LP (=2) | 2% lovastatin + 2% cholesterol ointment (compounded), applied BID | C/P/Poor: 1/4/0 | 3 months | NR |
| Buhle 2022 (17) | 1 | LP | 2% lovastatin + 2% cholesterol cream | Partial | NR | NR |
| Albanell-Fernández 2023 (18) | 2 | PPt | 1% simvastatin + 2% cholesterol ointment (petrolatum base) | C/P/Poor: 0/2/0 | 26 months (Case 1), 24 months (Case 2) | NR |
| Blue 2021 (19) | 1 | LP with bony resorption | 2% lovastatin + 2% cholesterol ointment (compounded), | Complete at selected sites; overall partial | 5 months | NR |
| Maredia 2024 (20) | 1 | LP (generalized) | 2% lovastatin + 2% cholesterol lotion, BID | Partial | 5 months | NR |
| Nguyen 2024 (21) | 1 | PM | 2% lovastatin + 2% cholesterol ointment (compounded) | Near-complete | Near-complete clearance by day 40; relapse after 8-week off period, improved on maintenance | NR |
| Alakeel 2023 (22) | 1 | LP | 2% atorvastatin + 2% cholesterol ointment, BID | Poor/No response | 10 weeks of topical statin use (added after 10 weeks of isotretinoin) | NR |
| Tomsitz 2022 (23) | 7 | DSAP | 2% lovastatin + 2% cholesterol cream | C/P/Poor: 2/5/0 | 12 weeks | NR |
| Maronese 2021 (24) | 1 | DSAP | 2% lovastatin + 2% cholesterol galenic cream, BID | Partial | 8 weeks | NR |
| Jerjen 2020 (25) | 2 | DSAP | 2% simvastatin + 2% cholesterol cream, BID | C/P/Poor: 0/2/0 | 3 months | NR |
| Peng 2024 (26) | 1 | SFP | 5% atorvastatin + 4% cholesterol cream  vs  4% cholesterol cream alone (split-face) | Partial | 48 weeks | NR |
| Chen K 2025 (27) | 1 | LP (coexisting with CHILD syndrome) | 2% lovastatin + 2% cholesterol ointment, BID | Partial | 4 weeks | NR |
| Aerts 2023 (28) | 1 | DSAP | 2% simvastatin + 2% cholesterol ointment (compounded) | Partial | 24 weeks | ACD |
| Saleva‐Stateva 2020 (29) | 5 | LP | 2% simvastatin + 5% cholesterol ointment (one case increased simvastatin to 5%) | Poor/No response | 10 months (Case 1); NR for others | NR |
| Barrabès-Torrella 2024 (30) | 20 | DSAP | lovastatin 2% (n=7), simvastatin 2% (n=2), lovastatin 2% + cholesterol 2% (n=3), simvastatin 2% + cholesterol 2% (n=8). Applied once or twice daily. | C/P/Poor: 4/15/1 | 1 to 10 months | Erythema and burning sensation (n=1), leading to discontinuation. |
| Herrero-Ruiz 2024 (31) | 9 | DSAP (n=7), LP (n=1), PM (n=1) | 2% simvastatin cream (monotherapy), applied once daily | Partial. Aggregate-only series; improvement in 8/9; per-patient tiers unavailable | 12 months (median follow-up) | Erythema and pruritus (n=2). |
| Ugwu 2020 (32) | 1 | DSAP | 2% lovastatin ointment (also tested 2% cholesterol + 2% lovastatin and 2% cholesterol alone) | Complete | 6 weeks | NR |
| Ryan 2023 **‡** (33) | 1 | PP | 2% lovastatin + 2% cholesterol ointment, BID; preceded by nightly topical salicylic acid to papules (stopped before statin) | Partial | 19 months | NR |
| McFeely 2021 (34) | 1 | DSAP | 2% simvastatin + 2% cholesterol cream, applied daily | Complete | 6 months | NR |
| Raison-Peyron 2025 (35) | 1 | LP | 2% simvastatin + 2% cholesterol cream (BID) | Partial | 2 months | ACD to simvastatin, confirmed by patch testing. |
| Badea 2024 (36) | 1 | PPt | 2% lovastatin ointment (BID) | Poor | 3 months | NR |
| Gao 2025 **†** (37) | 1 | DSAP | 2% lovastatin + 2% cholesterol ointment (BID) after pretreatment with Fractional CO₂ laser (single session) | Partial | 2 weeks | NR |
| Janaani 2024 **†** (38) | 1 | DSAP | 2% atorvastatin cream (compounded from tablets), with acitretin for 1 month | Partial | 6 months | NR |
| Cabo 2024 (39) | 1 | DSAP | 2% simvastatin cream (monotherapy, once daily) | Partial | 6 months | NR |
| Sultan 2023 (40) | 1 | DSAP | 2% lovastatin + 2% cholesterol gel (once daily) | Complete | 2 months | NR |
| Diep 2022 & 2023 (41,42) | 1 | LP (bilateral) | 2% lovastatin + 2% cholesterol ointment (right arm, BID for 12 weeks) followed by 2% lovastatin monotherapy (left arm, BID for 12 weeks, ~1 year later) | Partial | 12 weeks each arm | NR |
| Tan 2024  (43) | 17 | DSAP | 2% lovastatin + 2% cholesterol cream, once or twice daily | C/P/Poor: 0/10/7 (no complete clearances; 10/17 partial improvement, 7/17 no obvious improvement at first/last on-treatment assessment) | Median treatment 19 months, mean 23 months (range 6–46 months) | NR |
| Lang 2025 (44) | 19 | DSAP | 2% simvastatin + 2% cholesterol cream, once or twice daily | C/P/Poor: 3/16/0 (best IGA 0 in 3 patients = complete; best IGA 1–3 with clear improvement in remaining 16 = partial; no true non-responders) | Up to 18 months of treatment/follow-up | Mild local eczema/pruritus in several patients; one additional patient outside the efficacy cohort had patch-test–confirmed ACD to simvastatin |
| Howe 2025 (45) | 1 | DSAP | 2% lovastatin + 2% cholesterol ointment, BID (field therapy) | Complete/near-complete (~90–95% reduction in total lesion count at 12 months; no flares) | ≈ 12 months continuous treatment | NR |
| Möller 2025 (46) | 1 | DSAP | 2% simvastatin + 2% cholesterol ointment, once daily | Complete (all lesions cleared within 5 weeks; later new lesions also cleared rapidly with short re-treatment cycles) | 5-week initial course plus intermittent short re-treatments | NR |

**†** Adjunct therapy used (potential confounder): fractional CO₂ pretreatment (Gao 2025) or short-course acitretin (Janaani 2024). These cases were included in the primary dataset and excluded in the adjunct sensitivity analysis (see Results §3.10).

‡ Prior therapy: non-statin topical used before statin initiation (not classified as adjunct).

Notes. Outcome coding uses the review’s harmonized categories: C = complete/near-complete, P = partial, Poor = poor/no response (see Methods). Percent concentrations are w/w as reported; “compounded” indicates extemporaneously prepared formulations.

Abbreviations: ACD, allergic contact dermatitis; AEs, adverse events; BID, twice daily; DSAP, disseminated superficial actinic porokeratosis; IGA, Investigator’s Global Assessment; LP, linear porokeratosis; NR, not reported; PM, porokeratosis of Mibelli; PP, punctate porokeratosis; PPPD, porokeratosis palmaris et plantaris disseminata; PPt, porokeratosis ptychotropica; SFP, solar facial porokeratosis.
